# Primary pulmonary hyalinizing clear cell carcinoma with fusions of both EWSR1::CREM and IRF2::NTRK3: report of a case with an aggressive behavior

**DOI:** 10.3389/fonc.2023.1175279

**Published:** 2023-05-17

**Authors:** You-Li Wu, Feng Wu, Mian-Fu Cao, Yang Lan, Ming-Shan Du, Song-Tao Yu, Yan Wang, Xiao-Chu Yan, Xiu-Wu Bian, Guang-Jie Duan

**Affiliations:** ^1^ Institute of Pathology and Southwest Cancer Center, Southwest Hospital, Third Military Medical University (Army Medical University), Chongqing, China; ^2^ Department of Radiology, Southwest Hospital, Third Military Medical University (Army Medical University), Chongqing, China; ^3^ Department of Oncology, Southwest Hospital, Third Military Medical University (Army Medical University), Chongqing, China

**Keywords:** hyalinizing clear cell carcinoma, lung biopsy, EWSR1::CREM fusion, next-generation sequencing, prognosis

## Abstract

Primary pulmonary hyalinizing clear cell carcinoma (HCCC) is a rare salivary gland-type tumor newly recognized in recent years, with approximately 21 cases reported to date in the English literature, which constitutes a challenge in pathology diagnosis, particularly in small biopsy specimens. Here, we present a case of pulmonary HCCC diagnosed by computed tomography-guided percutaneous lung biopsy in a 70-year-old man’s right lower lung. Although the morphology and immunophenotype of the tumor suggested the diagnosis of mucoepidermoid carcinoma, fluorescence *in situ* hybridization failed to reveal the rearrangement of MAML2 gene, which is characteristic of mucoepidermoid carcinoma. Instead, further molecular genetic testing showed that the tumor harbored a rare EWSR1::CREM fusion combined with a previously unreported IRF2::NTRK3 fusion. Pulmonary HCCC is commonly regarded as a low-grade malignant tumor with an indolent course, but this case has a different biological behavior, presenting extensive dissemination and metastases at the time of diagnosis, which expands our understanding of the prognosis of this tumor. The patient has had five cycles of combination chemotherapy and has been alive with the tumor for eight months.

## Introduction

Primary pulmonary hyalinizing clear cell carcinoma (HCCC) is a rare new entity listed in the 2021 World Health Organization classification of thoracic tumors, which was first described by García et al. in 2015 (1), and only 21 cases have been reported in English publications to date (2–13). The typical histopathologic features of pulmonary HCCC are similar to those of the salivary gland counterparts, mainly composed of clear cells arranged in nests, cords, and trabecular patterns with hyalinization of the stroma. However, because of small tissue samples often with artificial extrusion in the preoperative biopsy, the morphology of pulmonary HCCC is variable and usually not typical, and the diagnosis of which remains very challenging.

We herein report a case of pulmonary HCCC diagnosed by computed tomography (CT)-guided percutaneous lung biopsy, the morphology and immunophenotype of which were similar to those of mucoepidermoid carcinoma. However the molecular genetic testing revealed that the tumor harbored a rare EWSR1::CREM fusion combined with an IRF2::NTRK3 fusion, rather than the rearrangement of MAML2 gene, which is characteristic of mucoepidermoid carcinoma (14). Meanwhile, we reviewed 21 cases reported previously (**Table 1**) to explore their clinicopathologic and imaging features, so as to strengthen our understandings of this rare tumor and, to improve diagnostic accuracy.

**Table 1:** Summaries of 22 cases of primary pulmonary hyalinizing clear cell carcinoma.

Case No.	Age (y)/Sex	Maximal Tumor Diameter (cm)	Location	Gross pathological features	Initial diagnoses	Molecular results	Treatment	Follow-up (mo)
1 (1)	38/M	2.6	Bronchus	Clear boundary	HCCC	EWSR1::ATF1 gene fusion	Surg	NED (10)
2 (2)	32/M	1.8	Segmental bronchus	Clear boundary, protrusion into the bronchi	SCC or low-grade MEC	EWSR1 gene rearrangement	Surg	NED (18)
3 (2)	39/M	2.6	Right lower lobe	Clear boundary	SCC or low-grade MEC	EWSR1 gene rearrangement	Surg	NED (18)
4 (3)	69/M	NA	Right upper lobe	NA	Lung cancer	EWSR1::ATF1 gene fusion	Surg + LND	AWD, with multiple pulmonary and lymph node metastases (192)
5 (4)	54/F	3.2	Left upper lobe	Clear boundary	SCC	EWSR1::ATF1 gene fusion	Surg	NED (16)
6 (5)	55/M	2.5	Right intermedius bronchus	Clear boundary, protrusion into the bronchi	NSCLC	EWSR1 gene rearrangement	Surg	NED (20)
7 (6)	75/F	0.9	Lower lobe	Clear boundary	MEC	EWSR1::CREM gene fusion	Surg	NED (8)
8 (7)	52/F	3.3	Segmental bronchus	Clear boundary, protrusion into the bronchi	HCCC	EWSR1::ATF1 gene fusion	Surg	NED (181)
9 (7)	35/F	2.8	secondary bronchus	Clear boundary, protrusion into the bronchi	HCCC	EWSR1::ATF1 gene fusion	Surg	NED (79)
10 (7)	56/F	3.3	secondary bronchus	Clear boundary, protrusion into the bronchi	HCCC	EWSR1::ATF1 gene fusion	Surg	NED (12)
11 (8)	66/F	1.3	Trachea	Polypoid mass	HCCC	EWSR1 gene rearrangement	Transbronchial laser resection	NA
12 (9)	55/F	2.5	Distal tracheal	Polypoid mass	HCCC	EWSR1 gene rearrangement	Surg + LND + RT	NED (72)
13 (10)	46/F	NA	Trachea	NA	SCC	EWSR1::ATF1 gene fusion	Surg + LND + ChT + RT	Recurrences (24), STD (72)
14 (11)	57/F	2.8	Right lower lobe	Clear boundary	MEC	EWSR1::ATF1 gene fusion	Surg + LND	AWD, with lymph node metastasis at diagnosis (3)
15 (12)	58/M	4.3	Right upper lobe	Clear boundary	Squamous papillary neoplasm	EWSR1::ATF1 gene fusion	Surg + ChT	NED (10)
16 (12)	60/F	2.0	Left lower lobe	Polypoid mass	HCCC	EWSR1::ATF1 gene fusion	Surg	NED (10)
17 (13)	44/M	3.5	Left lower lobar bronchus	NA	HCCC	EWSR1 gene rearrangement	Surg	NED (48)
18 (13)	56/F	1.6	Left upper lobar bronchus	NA	HCCC	EWSR1 gene rearrangement	Surg	NA
19 (13)	44/F	1.3	Right upper lobar bronchus	NA	HCCC	EWSR1 gene rearrangement	Surg	NA
20 (13)	33/F	4.9	Right middle lobar bronchus	NA	HCCC	EWSR1 gene rearrangement	Surg	NA
21 (13)	64/M	4.9	Left upper lobe hilum	NA	HCCC	EWSR1 gene rearrangement	Surg	NED (9)
22	70/M	5.3	Right lower lobe	Clear boundary	MEC	EWSR1::CREM gene fusion	ChT	AWD, with intrapulmonary, pleural, multiple bone and lymph node metastases (8)

22, Present case; M, male; F, female; NA, not available; HCCC, hyalinizing clear cell carcinoma; SCC, squamous cell carcinoma; MEC, mucoepidermoid carcinoma; NSCLC, non-small-cell lung carcinoma; Surg, surgery; LND, lymph node dissection; RT, radiotherapy; ChT, chemotherapy; NED, no evidence of disease; AWD, alive with disease; STD, succumbed to disease.

## Case presentation

**Figure 1:**
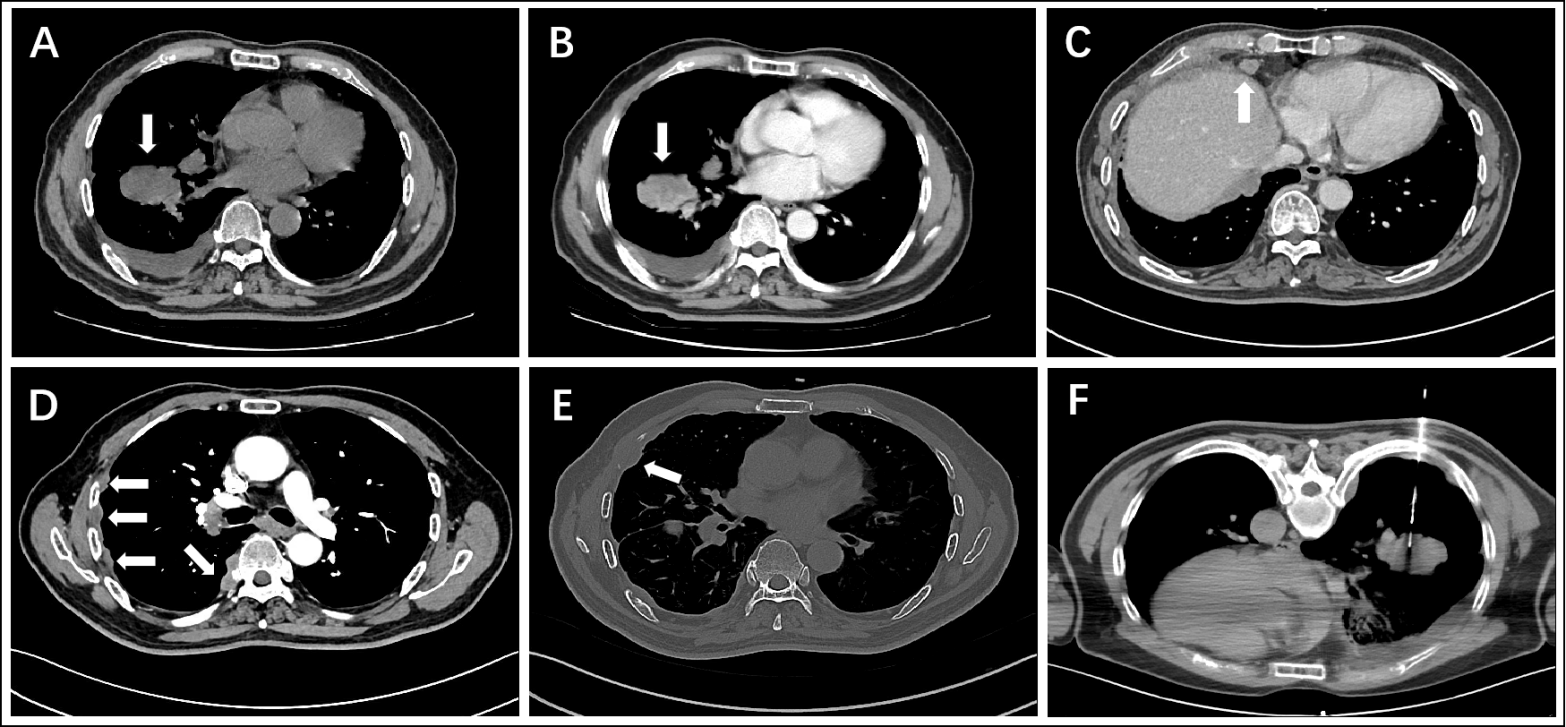
CT images show a lobulated mass in the right lower lobe of the lung **(A)** non-contrast, **(B)** contrast-enhanced), enlarged anterior supradiaphragmatic lymph nodes **(C)**, multiple thickening nodules in the right pleura **(D)**, bone destruction in the right fourth rib **(E)**, and CT-guided percutaneous lung biopsy **(F)**.

A 70-year-old male patient suffered from persistent dull pain in the right chest for two months, which was exacerbating at night. He presented neither symptoms of fever, cough, sputum, hemoptysis and dyspnea, nor history of chronic disease, infection, trauma, smoking and drinking. Chest CT revealed a lobulated mass with a diameter of approximately 5.3 cm in the right lower lobe of the lung close to the bronchus (**Figure 1A**), which was heterogeneously enhanced (**Figure 1B**). Multiple nodules were found in the middle and lower lobes of the right lung and the right interlobular fissure. CT also demonstrated multiple enlarged lymph nodes in the right subphrenic and anterior supradiaphragmatic spaces (**Figure 1C**), nodular thickening of the right pleura and the diaphragm around the liver (**Figure 1D**), and bone destruction in the right third, fourth, seventh, and eighth ribs (**Figure 1E**). All the findings suggested a lung cancer with metastases to intrapulmonary, pleural, diaphragm, right ribs and multiple lymph nodes. Subsequently, a CT-guided percutaneous lung biopsy was performed for pathological diagnosis (**Figure 1F**).

**Figure 2:**
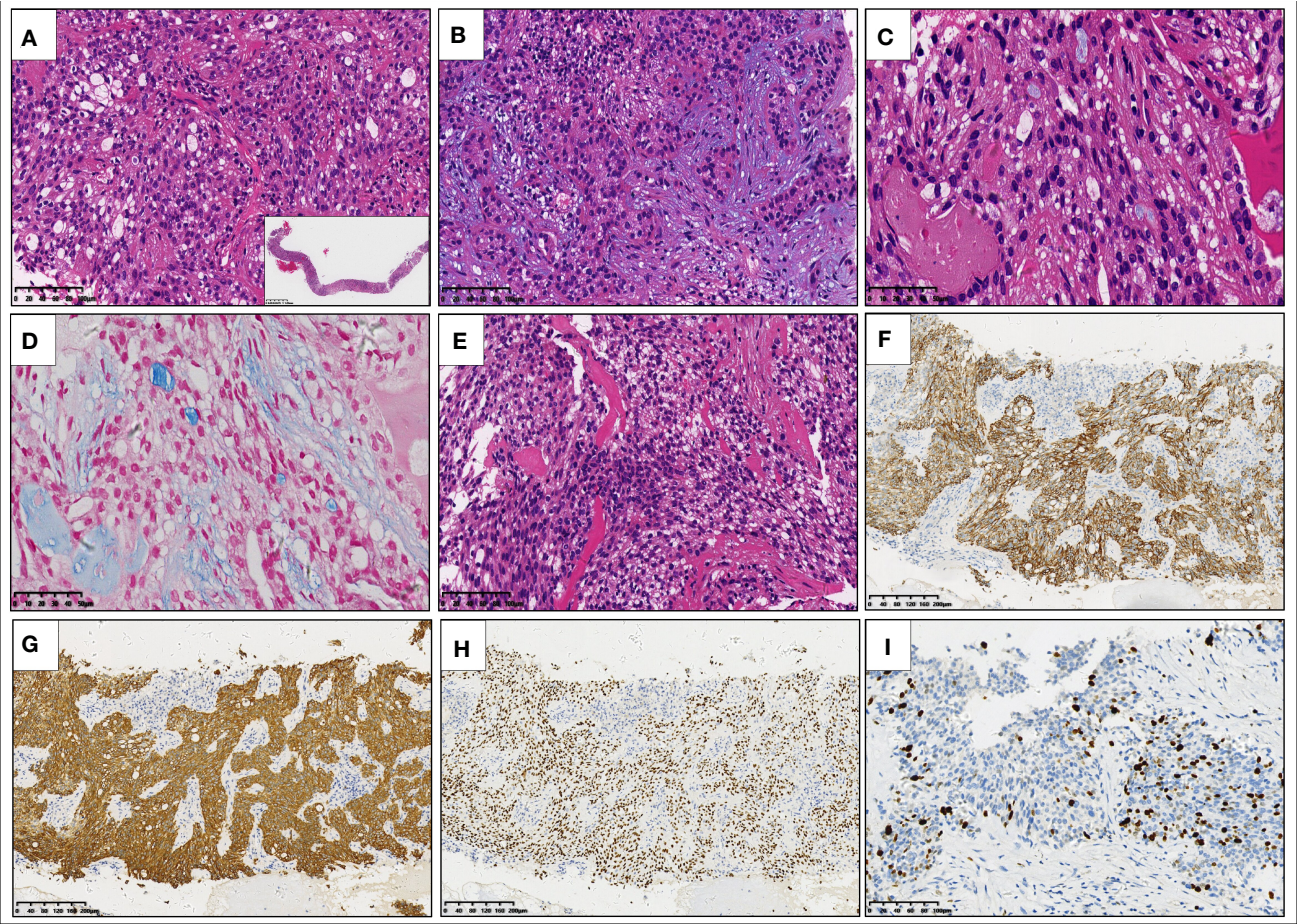
Hematoxylin and eosin staining of the core needle biopsy specimen reveals that epithelioid tumor cells with eosinophilic cytoplasm are arranged in nests **(A)** inset: low-power view of needle biopsy), cords, and trabeculae **(B)**. There are scattered mucocytes among the tumor cell nests **(C)**. Alcian blue staining highlights the mucocytes and loose myxoid stroma **(D)**. Cord-like collagen fibers are seen only in focal areas **(E)**. Immunohistochemical staining shows that the tumor cells are positive for CK5/6 **(F)**, CK7 **(G)**, and p40 **(H)**, the Ki-67 proliferation index of which is about 20% **(I)**.

Microscopically, the epithelioid tumor cells were arranged in nests (**Figure 2A**), cords, and trabeculae (**Figure 2B**), with moderate cytologic atypia. Most atypical cells had eosinophilic cytoplasm, except for some scattered tumor cells containing clear cytoplasm. Focally, the tumor showed cyst formation. The nuclei were round or oval with fine chromatin and small nucleoli. Neither significant mitotic activity nor necrosis was observed. Loose myxoid stroma and scattered mucus-secreting cells (mucocytes) were observed in focal areas (**Figure 2C**). The mucinous differentiation was demonstrated *via* Alcian blue staining (**Figure 2D**). Cord-like collagen fibers were observed only in focal areas (**Figure 2E**). Immunohistochemical staining showed that the tumor cells were positive for CK5/6 (**Figure 2F**), CK7 (**Figure 2G**), p40 (**Figure 2H**) and p63, but negative for S-100, SOX10, SMA, Calponin, and TTF1. The Ki-67 proliferation index was approximately 20% (**Figure 2I**).

**Figure 3:**
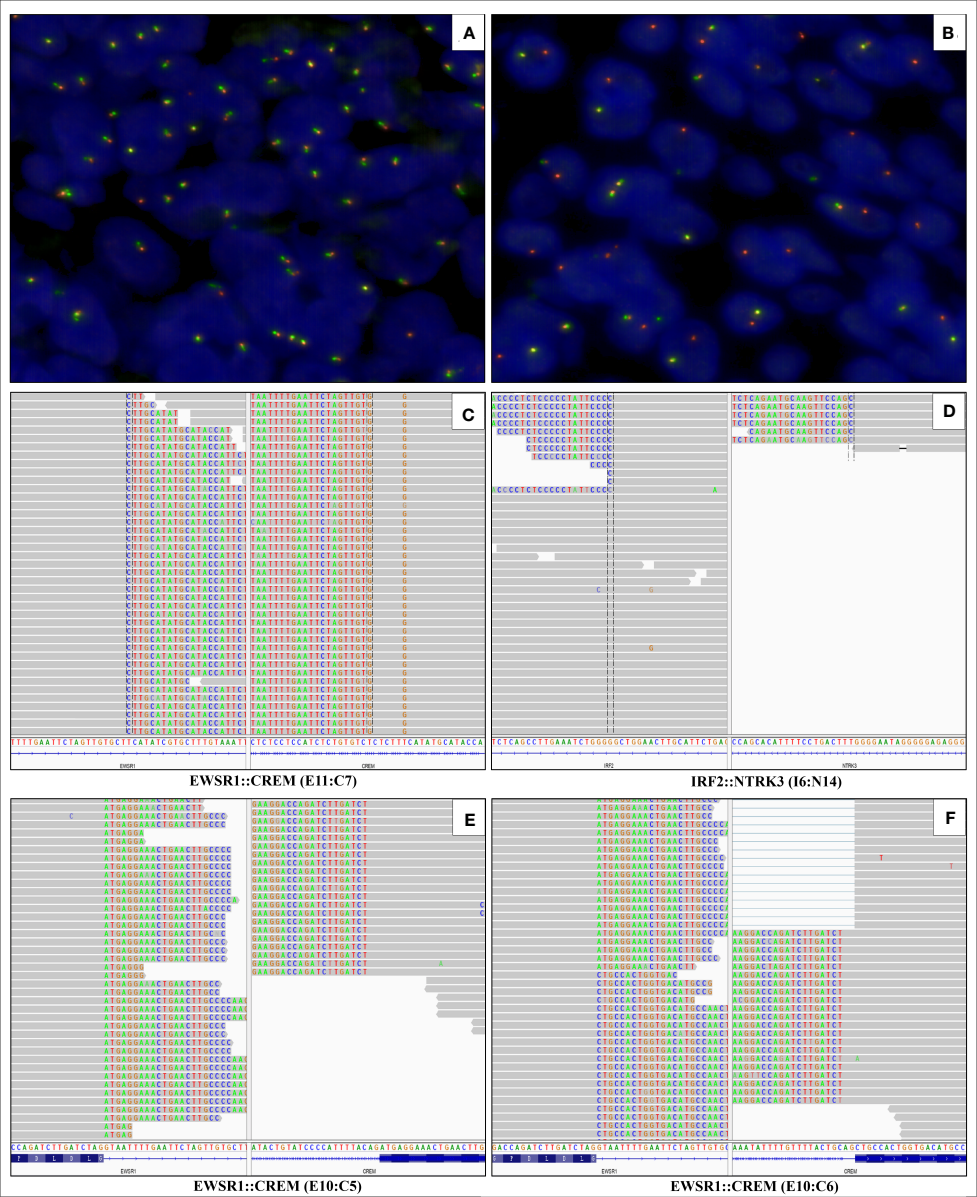
Fluorescence *in situ* hybridization is negative for MAML2 gene rearrangement **(A)** and positive for EWSR1 gene rearrangement **(B)**. Next-generation sequencing with a 425-gene panel reveals the EWSR1::CREM fusion (fusion of EWSR1 exon 11 to CREM exon 7) and IRF2::NTRK3 fusion (fusion of IRF2 intron 6 to NTRK3 intron 14) at DNA level **(C, D)**. RNA sequencing detects the EWSR1::CREM fusion transcript in which exon 10 of EWSR1 is fused to exon 5 and 6 of CREM **(E, F)**, respectively.

Although the morphology and the immunophenotype suggested the diagnosis of mucoepidermoid carcinoma (MEC), fluorescence *in situ* hybridization (FISH) failed to disclose the rearrangement of MAML2 gene (**Figure 3A**), a typical genetic alteration in MEC, which casted doubt on the initial diagnosis of MEC. It has been found in pathological practice that the pathological features of MEC resemble those of hyalinizing clear cell carcinoma (HCCC) or myoepithelial carcinoma. However, HCCC and myoepithelial carcinoma are featured with the rearrangement of EWSR1 gene, whereas MEC is not. Therefore, we performed FISH for the EWSR1 gene. Interestingly, we found the rearrangement of EWSR1 gene (**Figure 3B**), implying the nature of HCCC or myoepithelial carcinoma. Subsequently, next-generation sequencing (NGS) with a 425-gene panel was conducted, which revealed EWSR1::CREM fusion (**Figure 3C**) and IRF2::NTRK3 fusion (**Figure 3D**) at the DNA level. To determine whether there were potential targets of the NTRK inhibitor, newly approved by National Medical Products Administration (NMPA), the tissue sample was subjected to RNA sequencing (RNA-Seq). Unexpectedly, only EWSR1::CREM fusion was found at the RNA level (**Figures 3E, F**). The molecular profiling data indicated that the NTRK3 fusion gene encoded no protein product, and thus the patient could not receive the NTRK-targeted therapy. Surgery was not possible because the patient had substantial metastases and dissemination at the time of diagnosis. As a result, five cycles of combination chemotherapy were administered, and the patient has been alive with the tumor for eight months.

## Discussion

Primary pulmonary HCCC is a very rare salivary gland-type tumor recognized in recent years, with approximately 21 cases so far reported in the English literature (1–13). Together with our newly identified HCCC case, the 22-case cohort (**Table 1**) demonstrates that it mainly occurred in middle-aged and older adults, with ages ranging from 32 to 75 years (median, 55 years; average, 53 years). The ratio of male to female patients was 9:13. Most patients lacked specific clinical manifestations and apparent symptoms. The tumor masses in the lung were often observed during regular physical examinations. A few patients presented back pain, chest pain, cough, hemoptysis, and dyspnea. Some patients had a history of smoking. The maximal diameter of the tumor ranged from 0.9 cm to 5.3 cm, and our case was the largest one reported so far.

Imaging presents that most HCCCs are well-demarcated, and destruction of the surrounding bronchus is recognized in some cases. Unlike common lung cancers, primary pulmonary HCCC is often close to and grows along the bronchus. These imaging features of HCCC may be related to its origin from the bronchial submucosal glands (7). CT and fiberoptic bronchoscopy also find some polypoid masses in the bronchial lumen, which may lead to airway obstruction. Icard et al. reported a case of HCCC undergoing emergency admission due to progressive dyspnea. Fiberoptic bronchoscopy unveiled a polypoid mass blocking 60% of the tracheal lumen, and emergency endobronchial laser resection was performed to relieve the obstruction (8). Therefore, clinicians should pay attention to the acute airway events in this disease.

The patient herein is the first case of HCCC diagnosed by CT-guided percutaneous lung biopsy. In the previously described cases, 20 of 21 (95%) were diagnosed after surgical resection, and only one was diagnosed by bronchoscopic biopsy (11). Jeffus et al. reported a case initially misdiagnosed as moderately to poorly differentiated squamous cell carcinoma (SCC) with a biopsy specimen (4). Based on the bronchoscopic biopsy specimen, two cases were misdiagnosed as non-small cell lung cancer and squamous papillary neoplasm, respectively (5, 12). The postoperative cases were also often diagnosed as other common tumors, such as MEC (1 case), SCC (2 cases), SCC/MEC (2 cases), and lung cancer (1 case) (2–4, 6, 10), which indicates a high misdiagnosis rate of pulmonary HCCC.

Together, the diagnostic difficulty might be attributed to the followings: (a) The morphology of HCCC is variable. The tumor cells might contain clear or eosinophilic cytoplasm, and the hyalinizing stroma might not be apparent. Additionally, the tumor cells might present with squamous and mucinous cell differentiation, pathologically resembling MEC, SCC, and myoepithelial carcinoma; (b) HCCC lacks specific immunohistochemical markers, and its immunophenotype overlaps with MEC, SCC, and myoepithelial carcinoma; and (c) Most importantly, primary pulmonary HCCC is so scarce that both clinicians and pathologists do not have enough awareness and understanding of this new entity.

It is challenging to distinguish HCCC from MEC because both show epidermoid and mucinous differentiation. Among the 22 cases, 5 (23%) were initially diagnosed as MEC (2, 6, 11). MEC is the most common malignant salivary gland-type lung tumor, and is more aggressive than HCCC. Our case had been initially diagnosed as a stage IV lung cancer with high invasiveness, which prompted us to consider this case as MEC. However, the lack of MAML2 gene rearrangement and the acquisition of EWSR1 gene rearrangement argued against the diagnosis of high-grade MEC. Therefore, the characteristic molecular changes are crucial in the differential diagnosis. Pulmonary HCCC should also be distinguished from SCC. Among 22 cases, 4 cases (18%) were initially considered as SCC (2, 4, 10). Compared with HCCC, SCC has remarkable cellular atypia with prominent nucleoli and obvious mitotic figures. Although both HCCC and SCC consistently express CK5/6, CK7, and p40, the Ki-67 proliferation index of HCCC is usually lower than that of SCC. Therefore, if the Ki-67 proliferation index is relatively low, the possibility of HCCC should be considered.

Pulmonary HCCC has characteristic molecular changes, including EWSR1::ATF1 fusion (10 of 11 cases) and EWSR1::CREM fusion (1 of 11 cases) (1–13). In our case, NGS revealed a rare EWSR1::CREM fusion and an unreported IRF2::NTRK3 fusion at the DNA level. Although the gene rearrangement of EWSR1 can occur in both HCCC and myoepithelial carcinoma, the fusion partners are different. Myoepithelial carcinoma often presents with EWSR1::PBX1 fusion, EWSR1::ZNF444 fusion, or FUS::KLF17 fusion. Therefore, NGS, RNA-seq, or other methods of fusion gene detection could be used for differential diagnosis.

Complete resection of the mass is the first choice for treating pulmonary HCCC (21/22 cases). It remains unclear whether regional lymph node dissection or postoperative chemoradiotherapy is necessary. In our case, the patient received five cycles of combined chemotherapy (paclitaxel plus nedaplatin). However, the CT showed that the primary tumor and metastatic lesions had no response after chemotherapy. The chemotherapy will be continued, and the therapeutic effect will be further followed up.

Primary pulmonary HCCC is commonly considered a low-grade malignant tumor with an indolent clinical course. Analysis of 18 patients with an average follow-up of 44 months revealed that 14 (78%) survived without tumors, four experienced disease relapses or metastases, and only one died. However, in our case, the patient had active disease progression with extensive dissemination and metastases at the time of diagnosis, which is different from those reported in the literature and expands our understanding of the biological behavior of the pulmonary HCCC. Specifically, the case had unusual genetic changes including EWSR1::CREM fusion and IRF2::NTRK3 fusion, and the largest tumor diameter of 5.3 cm, whether these phenotypes were associated with poor prognosis of the patient remained to be investigated.

## Conclusion

The diagnosis of primary pulmonary HCCC is very challenging because of its rarity, particularly in small biopsy specimens. This paper reports an unusual pulmonary HCCC diagnosed by CT-guided percutaneous lung biopsy. Further understanding of this enigmatic tumor is essential to augment its diagnostic accuracy.

## Data availability statement

The original contributions presented in the study are included in the article/supplementary material. Further inquiries can be directed to the corresponding author.

## Ethics statement

Written informed consent was obtained from the individual(s) for the publication of any potentially identifiable images or data included in this article.

## Author contributions

Material preparation, data collection and analysis were performed by Y-LW, YL, M-SD, and S-TY. The first draft of the manuscript was written by Y-LW, FW, and M-FC. The manuscript was revised and reviewed by G-JD, X-WB, X-CY, and YW. All authors contributed to the article and approved the submitted version.
